# Intervention methods and influencing factors of vitamin D deficiency in Northeast China: a self-controlled trial and randomized controlled trial study

**DOI:** 10.3389/fnut.2026.1778526

**Published:** 2026-08-03

**Authors:** Junyi Liu, Chenyin Qu, Shanshan Jin, Yubing Jia, Lixin Na

**Affiliations:** 1 Department of Clinical Nutrition, Shanghai University of Medicine and Health Sciences Affiliated Zhoupu Hospital, Shanghai, China; 2 Department of Nutrition and Food Hygiene, Public Health College, Harbin Medical University, Harbin, China; 3 College of Public Health, Shanghai University of Medicine and Health Sciences, Shanghai, China; 4 Department of Nutrition, Chengdu Women's and Children's Central Hospital, School of Medicine, University of Electronic Science and Technology of China, Chengdu, China; 5 Department of Clinical Nutrition, Chongqing Hospital of Jiangsu Province Hospital, Chongqing, China

**Keywords:** children, single nucleotide polymorphisms, ultraviolet irradiation, vitamin D deficiency, vitamin D supplementation

## Abstract

**Background:**

Vitamin D deficiency is common among children in high-latitude regions. However, region-specific intervention strategies remain unclear. Few studies have simultaneously examined outdoor activity intervention, vitamin D supplementation, and genetic polymorphisms. Thus, this study aimed to explore feasible intervention strategies for children in Northeast China.

**Method:**

Sixty-one children were asked to increase outdoor activities for 8 weeks (June–August). Outdoor activity duration and ultraviolet (UV) intensity were recorded. Changes in serum 25(OH)D_3_ levels and the association with UV exposure were analyzed. Separately, 289 children were randomized to receive 0, 400, or 800 IU/d vitamin D for 8 weeks. The dose-response relationship between vitamin D intake and serum 25(OH)D_3_ was analyzed. Effects of *CYP2R1* single nucleotide polymorphisms (SNPs) (rs1562902, rs10741657) on supplementation response were also evaluated.

**Results:**

Outdoor activity duration increased by 11 min/d, and estimated vitamin D production from UV increased by 60.73 IU/d, but serum 25(OH)D_3_ did not change significantly (18.52 ± 4.07 vs. 18.25 ± 3.11 ng/ml); this change was not associated with outdoor time, UV intensity, or calculated vitamin D production from UV. In the supplementation trial, serum 25(OH)D_3_ increased by 8.82 ± 7.53 ng/ml in the 400 IU/d group and 15.06 ± 9.40 ng/ml in the 800 IU/d group. Total vitamin D intake (diet + supplement) was estimated to require at least 733.21 IU/d to achieve serum 25(OH)D_3_ ≥30 ng/ml. Children with CT/TT genotypes (rs1562902) and AG/GG genotypes (rs10741657) showed a trend of lower sensitivity to supplementation.

**Conclusion:**

Increasing outdoor activity did not improve vitamin D status in children in Northeast China, but daily supplementation was effective. Dietary vitamin D intake and SNPs should be considered when formulating individualized supplementation doses.

## 1 Introduction

Vitamin D deficiency is a global public health problem, especially among the elderly, pregnant women and children (1–3). It is reported that the prevalence of vitamin D deficiency and insufficiency among children exceeds 50%, which is more severe in developing countries and regions (4–8). In addition to being related to bone health, vitamin D deficiency is associated with extra-skeletal health, such as obesity, insulin resistance, cardiovascular disease, beta-cell dysfunction, autoimmune diseases, respiratory tract infection and cancer (9, 10). Furthermore, vitamin D deficiency in childhood has been found to affect health even in adulthood, such as increasing the risk of cardiovascular and metabolic diseases (11). Therefore, ensuring adequate vitamin D supply in children is important, and has become a focus of current research.

Studies have shown that 90% of the body's vitamin D supply is synthesized within the skin (12). A study conducted in New Zealand and Australia showed that spending 5–10 min outdoors between 8 a.m. and 4 p.m. during summer, with 35% of the body surface area exposed to sunlight, is sufficient to maintain existing 25(OH)D concentrations (13). However, the association of serum 25(OH)D_3_ with natural UV exposure is limited. In addition, UV irradiation is usually affected by the season, latitude and other factors. People who live in latitudes >35 °N have been found to have an increased risk of vitamin D deficiency (14, 15). The incidence of vitamin D deficiency was higher in winter than in summer, because less UV could reach the earth in winter (16). Lifestyle can also affect vitamin D status: decreased outdoor activities and the use of sun protection are likely to increase the risk of vitamin D deficiency (17, 18). So far, there have been very few studies specifically exploring the intervention effect of increasing outdoor activities on vitamin D deficiency in children. Whether enhancing natural UV exposure can become an effective and scalable intervention method for vitamin D deficiency in children still requires further research.

At present, countries vary in their standards for the dosage of vitamin D supplementation for children. Rebecca J Moon considered that children in the UK with a high risk of vitamin D deficiency should supplement at least 400 IU/d of vitamin D to reduce the risk of rickets or hypocalcaemia (19). Mario Flores-Aldana's study found that vitamin D_3_ supplementation of 1,000 IU/d for 3 months was effective in reducing vitamin D deficiency in children aged 12 to 30 months (20). A study conducted in Mongolia has shown that weekly supplementation with 140,000 IU of vitamin D3 for three consecutive years can effectively increase vitamin D levels in children with deficiency (21). Therefore, if vitamin D supplementation is to be adopted, appropriate supplementation plans need to be developed for children from different regions and ethnic groups.

Not only diet and UV exposure but also genetic factors, especially single nucleotide polymorphisms (SNPs), are related to serum 25(OH)D_3_ concentration (22–24). Studies demonstrated that people with vitamin D deficiency had a higher frequency of AG and GG genotypes in cytochrome P450 2R1 (*CYP2R1*) rs10741657 and AC and CC genotypes in vitamin D binding protein (*VDBP*) rs2282679 (25). Tomei S's study showed that both *VDR* rs731236 and *CYP2R1* rs7116978 had a significant association with serum 25(OH)D concentration, where the rs731236 AA and AG and the rs7116978 TT and CT genotypes were associated with vitamin D deficiency (26). These findings suggest that the influence of SNPs should be taken into account when assessing vitamin D nutritional status or intervention effects.

In our previous study, we investigated the vitamin D nutritional status of children in Harbin, located in Northeast China at latitudes ranging from 44 °N to 46 °N. Compared with southern regions of China, this area features a higher latitude, a lower ultraviolet radiation intensity and a longer winter season, with relatively few vitamin D-rich foods in the local regular diet. Only 10% of children had sufficient vitamin D levels (27). However, studies focusing on vitamin D status among children in Northeast China remain limited, and the methods employed in these studies are rather simplistic. This study adopted a combined strategy of outdoor activity and vitamin D supplementation, while considering the potential impact of single nucleotide polymorphisms, to explore effective approaches for improving vitamin D status among children in Northeast China.

## 2 Methods

### 2.1 Subject

**Figure 1:**
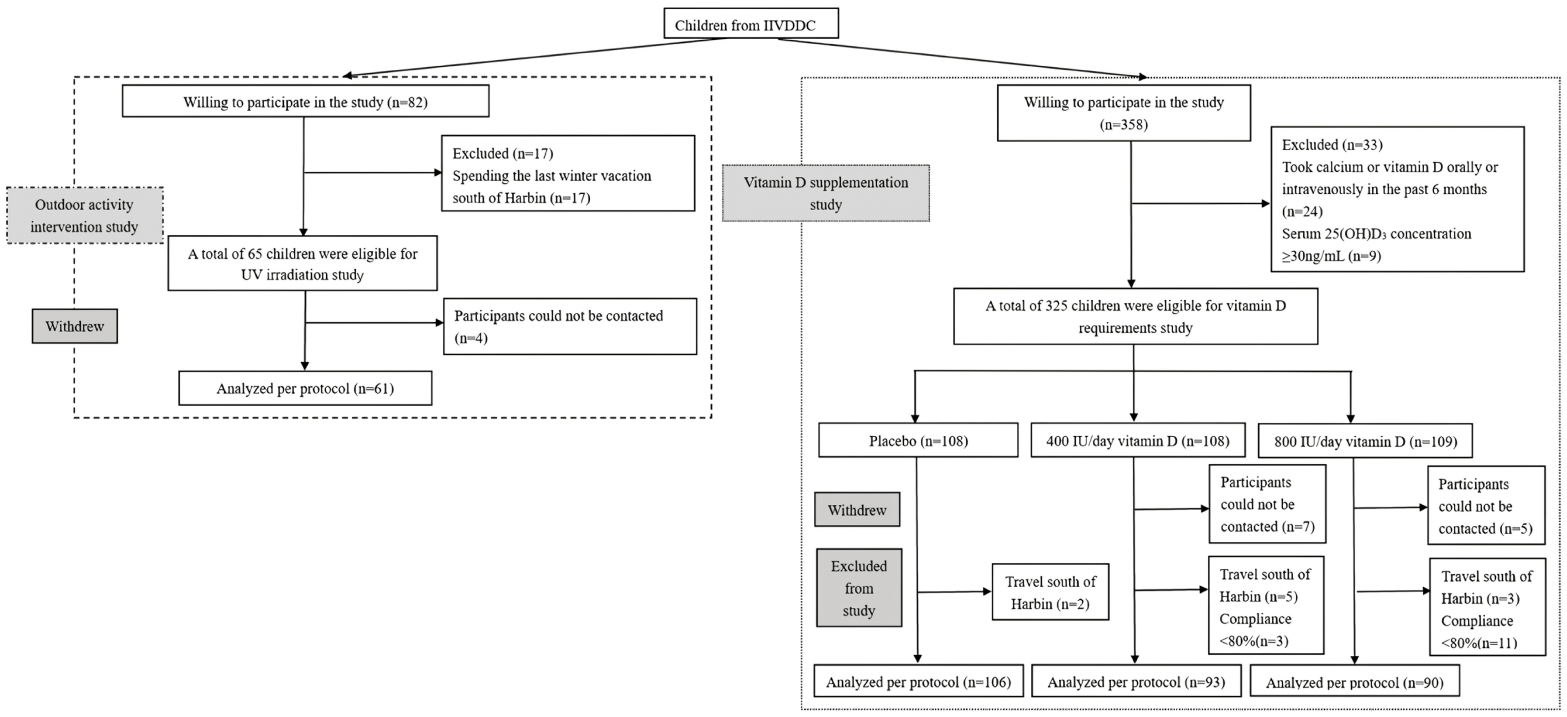
Flow diagram of subject enrollment and random assignment to and analysis of study intervention groups.

There were two trials: an outdoor activity intervention and a vitamin D supplementation trial. All the participants were from the project of Investigation and Intervention of Vitamin D Deficiency in Children (IIVDDC). Four kindergartens (2 public, 2 private) in Nangang District of Harbin were selected by convenience sampling according to different economic levels. From these kindergartens, all eligible children were recruited. The formula for calculating the sample size is as follows: $n=\frac{{2\left(Z_{1-\alpha /2}+Z_{1-\beta }\right)}^{2}\sigma^{2}}{\delta^{2}}$. Among them, the bilateral test significance level α = 0.05, the test power 1—β = 0.80, Z_0.975_ = 1.96, and Z_0.80_ = 0.84. Based on similar nutritional intervention studies, the standard deviation σ of serum 25(OH)D_3_ concentration was set at 10 ng/ml, and the minimum clinically significant mean difference δ between the two groups was set at 8 ng/ml. After calculation, each group required 25 cases, and the three groups totaled 75 cases. The parents and teachers of children were invited to informational meetings at which the study and its procedures were explained. A total of 440 children aged 3–7 years who had lived in Harbin for the past 3 years were eligible as participants. There were 82 subjects who were willing to participate in the outdoor activity intervention study. After excluding 17 children who spent their last winter holiday at a lower latitude than Harbin, 65 children received the outdoor activity intervention. During the intervention, 4 children could not be contacted. Finally, 61 participants completed the 8-week trial from June 2018 to August 2018 (Figure 1). There were 358 children who were willing to participate in the vitamin D supplementation study. After excluding 24 children who had taken calcium or vitamin D orally or intravenously in the past 6 months and 9 children with serum 25(OH)D_3_ concentration ≥30 ng/ml, the remaining 325 children received vitamin D supplementation. After intervention, 12 children could not be contacted, 10 children had a vacation during the intervention in an area with a lower latitude than Harbin and 14 children with compliance < 80%. Finally, 289 participants completed the 8-week intervention trial from September 2018 to November 2018 (Figure 1).

The methods in the study were in accordance with the approved guidelines and were approved by the ethics committee of Harbin Medical University. The registered ethical number: ChiCTR1800020294 for IIVDDC.

### 2.2 Study design and intervention

The outdoor activity intervention was a self-controlled trial lasting 8 weeks. Before the intervention, a questionnaire survey was conducted to collect basic information including diet and outdoor activity habits. During the intervention, subjects were asked to increase the duration of outdoor activities as much as possible during summer, when UV intensity is high and children can spend more time outdoors. Children in the outdoor activity intervention were asked not to use sun protection measures, including parasols, sun-protective clothing, and sunscreen, so as to ensure sufficient UV exposure. Guardians and teachers were reminded to record children's daily activity time, frequency, and whether to use sun protection measures. At the same time, researchers recorded the UV intensity at different time points of the day during the intervention, and calculated the amount of vitamin D produced under UV irradiation. After the intervention, the change in serum vitamin D concentration and its association with outdoor time, UV intensity and vitamin D produced from UV exposure were analyzed.

The vitamin D supplementation study was a single-blind randomized controlled trial. Subjects were allocated to take daily 400 IU, 800 IU vitamin D_3_ or placebo after breakfast for 8 weeks. Placebos were capsules containing vegetable oil that match the shape and taste of vitamin D_3_ supplements. Subjects were randomly grouped using a computer-generated block randomization procedure, and a continuous list of research identification numbers related to the supplementary doses was generated. Throughout the entire trial period, all subjects were blinded to the intervention assignments, while the staff knew the group allocations. Before the intervention, a questionnaire survey was conducted to collect basic information including diet and outdoor activity habits. During the intervention, children were asked to maintain their original living habits. Staff followed up regularly by phone to ask about the remaining number of capsules and reminded parents to give their children vitamin D capsules as required. At the end of the intervention, the remaining number of capsules for each child was counted. Total vitamin D intake was calculated and serum vitamin D concentration was measured after the intervention. Serum concentrations of vitamin D_3_, alkaline phosphatase (ALP) and parathyroid hormone (PTH) were determined before and after the intervention. The association of total vitamin D intake, including diet and supplements, with serum vitamin D_3_ concentration was analyzed. In addition, the association of changes in serum vitamin D_3_ after supplementation with vitamin D metabolism-related SNPs was also analyzed.

### 2.3 Data collection by the questionnaire

Detailed in-person interviews were administered by trained personnel using a structured questionnaire to collect information on demographic characteristics, outdoor activity, sun protection and dietary intake in both intervention studies.

Questionnaires for both studies were completed by parents and teachers in kindergartens together. The demographic characteristics of the children included age and gender. Subjects were rated for their sun protection methods, including four evaluation criteria, namely sun avoidance during outdoor activities, use of sunscreen, parasols, and sun-protective jackets. The points were graded from 0 to 3 based on the reported frequency: never (0), occasionally (1), often (2), and always (3). A total score ≤ 4 was classified as low sun protection, while a score >4 was considered high sun protection. Outdoor activity adherence was defined as good if daily outdoor activity time was ≥60 min, and poor if < 60 min. Dietary information was collected using a food-frequency questionnaire (FFQ) which had good reliability and validity. The correlation coefficients of major nutritional factors between FFQ1 and FFQ2 were 0.61–0.70 and major nutritional factors between FFQ1 and 3-day dietary record were 0.61–0.69, respectively (27). A total of 48 food items were included in the questionnaire, which covered most of the foods in the recipes of the kindergartens included in our study. For each food item, parents and teachers were asked how frequently participants consumed over the preceding year, followed by a question on the amount consumed in Liang (a unit of weight equal to 50 g) or ml (for liquid food item) per unit of time. The consumption frequency was transformed to obtain mean consumption per day. Nutrient intakes for each food item, except vitamin D, were calculated using the China Food Composition Tables.

### 2.4 Anthropometric measurement

Anthropometric measurements, including height and weight, were taken by well-trained examiners, with participants wearing light, thin clothing and no shoes. Body weight and height were measured to the nearest 0.1 kg, and 0.1 cm, respectively. Body mass index (BMI) was calculated as weight (kg) divided by the square of the height in meters (m^2^). Children's sex- and age-adjusted z-scores for body mass index (zBMI) were calculated according to the WHO growth references (28, 29) using the formula: zBMI = (child's BMI – median BMI of the reference population of the same age and sex)/standard deviation (28).

### 2.5 UVB exposure measurement

UV energy tester (UV–Integrator150, Speeder, Shenzhen, China) was used to measure UV exposure. The instrument was placed in an open and unshaded field outdoors to simulate the light pattern received by children during outdoor activities. According to the metrical data, the intensity of UV within a day was classified into three levels: UV intensity was highest from 10:00 to 12:00, medium from 9:00 to 10:00 and from 12:00 to 14:00, and lowest from 8:00 to 9:00 and after 14:00. The average UV exposure intensity of different periods was calculated, and the UV exposure dose received by children was calculated with the exposure time and skin area of children.

### 2.6 Calculation of vitamin D dose generated by UV radiation

Vitamin D production from UV irradiation was calculated by formulas from study of Maria-Antonia Serrano (30). According to Equation 1 below, the daily personal UV erythemal (UVER) dose in J/m^2^ was converted to vitamin D doses (UVD) in J/m^2^ using action spectrum conversion factors (ASCFs), which were function of the latitude, season of the year and ozone content of the atmosphere.


$$\begin{array}{l} UVD\left(J/m^{2}\right)=\;UVER\left(J/m^{2}\right)\cdot ASCF. \end{array}$$ (1)

Using the following Equation 2, the minimum vitamin D dose (MDD) for other skin types and body exposures was estimated, where percentage of body exposure (PBE) was the exposed body fraction, and the value of PBE in this study was 0.3. The skin type factor (STF) was used to adjust for skin types other than type III. In Equation 3, a minimal erythemal dose (MED) was the minimum UVER dose that caused erythema with sharply defined edges 24 h after sun exposure and its value depended on skin type.


$$\begin{array}{lll} MDD\left(J/m^{2}\right) & = & \frac{30\left(J/m^{2}\right)}{STF\cdot PEB} \end{array}$$ (2)

$$\begin{array}{lll} STF & = & \frac{250\left(J/m^{2}\right)}{MED\left(J/m^{2}\right)} \end{array}$$ (3)

Finally, Equation 4 was used to estimate the amount of daily vitamin D produced from a personal median daily UVER exposure:


$$\begin{array}{l} Vitamin\;D\left(IU/day\right)=\frac{UVD\left(J/m^{2}/day\right)\cdot 1000IU}{MDD\left(J/m^{2}\right)}\cdot \frac{AF}{SPF} \end{array}$$ (4)

AF is the age factor, different age groups have different AF values, and the children in this study had an AF value of 1.0 (31). SPF is the sun protection factor of any sun block applied over the entire exposed body surface. The sun protection factor (SPF) for children whose skin without protection was 1, and the SPF for children who use sun protective cream in summer was 30 (30).

### 2.7 Vitamin D from diet calculation

Vitamin D from diet was calculated based on the U.S. nutrient database (USNDB, National Nutrient Database for Standard Reference of the U.S. Department of Agriculture—USDA) (32) as well as the frequency of consumption of each food item from the FFQ.

### 2.8 Biochemical assessment

Fasting (more than 10 h) blood samples were collected from children in the IIVDDC. 25(OH)D_3_ was measured using ELISA kits (Mlbio, <city>Shanghai</city>, China. Catalog Number: ml 103901), with a detection wavelength of 450 nm, a limit of detection of 1.4 ng/ml, and the inter- and intra-assay coefficients of variation were 9.5% and 6.7%, respectively. Serum 25(OH)D_3_ < 10 ng/ml was considered severe deficiency, 10–20 ng/ml was considered deficiency, 20–29 ng/ml was considered insufficiency, and ≥30 ng/ml was considered sufficiency (33). ALP and PTH were measured using ELISA kits (Mlbio, <city>Shanghai</city>, China).

### 2.9 Selection and detection of SNPs

Ten candidate genes were selected according to the following criteria: (1) they had evidence of significant association with vitamin D in previous studies; (2) they had biological importance in vitamin D metabolism, transportation, degradation, or vitamin D signaling pathways. Finally, *CYP2R1* was selected as the gene of interest, and two SNPs in *CYP2R1* were selected, including rs1562902 and rs10741657.

For each subject, genomic DNA was extracted from peripheral blood using the Puregene DNA isolation kit following the provided protocol. DNA samples were diluted to 20 ng/μl and shipped to Oebiotech (<city>Shanghai</city>, China) for SNP genotyping using the Multiple PCR technology.

A 10 μl PCR reaction system was prepared. After a total of 50 cycles of denaturation, annealing, and extension, the PCR amplification product was obtained.

High-resolution melting analysis was performed on the PCR amplification product. The product was melted at 95 °C for 1 min, cooled at 40 °C for 1 min, and then slowly heated from 65 °C to 95 °C. During this process, fluorescence detection was conducted 25 times per 1 °C increase, and a high-resolution melting curve was obtained. When the melting curves of each sample were detected and analyzed, the time at which the DNA (marked with different colors) started to unwind was observed in real time, and the base type at each locus was determined according to differences in melting temperature.

Blank, positive, and duplicate controls were set for quality control. The genotyping success rate was 98.1%. About 5% of samples were randomly selected for consistency verification, with a consistency rate of 99.3%.

### 2.10 Statistical analysis

SPSS v22.0 (Beijing Stats Data Co. Ltd, Beijing, China) was used to analyze the data, and a two-sided *P* value < 0.05 was considered statistically significant. Continuous and categorical variables were presented as mean ± standard deviation and *n* (%), respectively. ANOVA, analysis of covariance (ANCOVA), and chi-square test were used to compare the differences in continuous variables and categorical variables among the groups. *Post hoc* Tukey's test was used for pairwise comparisons. ANCOVA was used to analyze the differences of serum 25(OH)D_3_ concentration among genetic polymorphism groups. The linear regression analysis was used to analyze the association of serum 25(OH)D_3_ concentration with UV exposure dose in children, presented as unstandardized and standardized β coefficients with their 95% confidence intervals. Quadratic regression analysis was used to analyze the association of serum 25(OH)D_3_ concentration with oral intake of vitamin D in children. Sensitivity analyses, including bootstrap internal validation (1,000 resamples) and multivariable linear regression adjusting for age, sex, and BMI, were performed to assess the robustness of the dose-response model.

## 3 Results

### 3.1 Results from outdoor activity intervention study

#### 3.1.1 Baseline characteristics of subjects

A total of 61 children were analyzed in this study, 41 of whom had serum 25(OH)D_3_ concentration < 20 ng/ml, representing about 67% of the total population. There were no significant differences in sex, age, height, weight, zBMI and energy intake between the two groups. In terms of sun protection habits, the two groups had distinct distributions (*P* = 0.030): the proportion of low sun protection was higher among children with serum 25(OH)D_3_ ≥20 ng/ml, whereas high sun protection was more common in those with serum 25(OH)D_3_ < 20 ng/ml (Table 1).

**Table 1:** Baseline characteristics of subjects^1^.

Variable	Serum 25(OH)D_3_ concentration < 20 ng/ml (*n* = 41)	Serum 25(OH)D_3_ concentration ≥20, ng/ml (*n* = 20)	*P*
Male, *n* (%)	19 (46.3)	12 (60.0)	0.316
Age, years	5.59 ± 1.81	5.32 ± 0.96	0.384
Height, cm	114.91 ± 14.63	111.74 ± 8.33	0.372
Weight, kg	22.32 ± 10.06	20.52 ± 4.56	0.582
zBMI	0.38 ± 1.36	0.60 ± 1.15	0.396
Energy, Kcal/d	1,327.98 ± 430.13	1,215.02 ± 300.33	0.296
Sun protection, *n* (%)			
Low sun protection	24 (58.5)	19 (95.0)^*^	0.030
High sun protection	17 (41.5)	1 (5.9)^*^	
Outdoor exercise period in summer, *n* (%)			
High UV period	21 (51.2)	10 (50.0)	0.996
Medium UV period	6 (14.6)	3 (15.0)	
Low UV period	14 (34.1)	7 (35.0)	
Outdoor activity duration, min/d	70.24 ± 30.45	70.50 ± 35.46	0.977

^1^Values are mean ± SD or *n* (%) for all variables. A two-sided *P* < 0.05 was considered statistically significant. ^*^P < 0.05 compared with serum 25(OH)D_3_ concentration < 20 ng/ml group.

#### 3.1.2 Detection of UV intensity during the outdoor activity intervention

**Figure 2:**
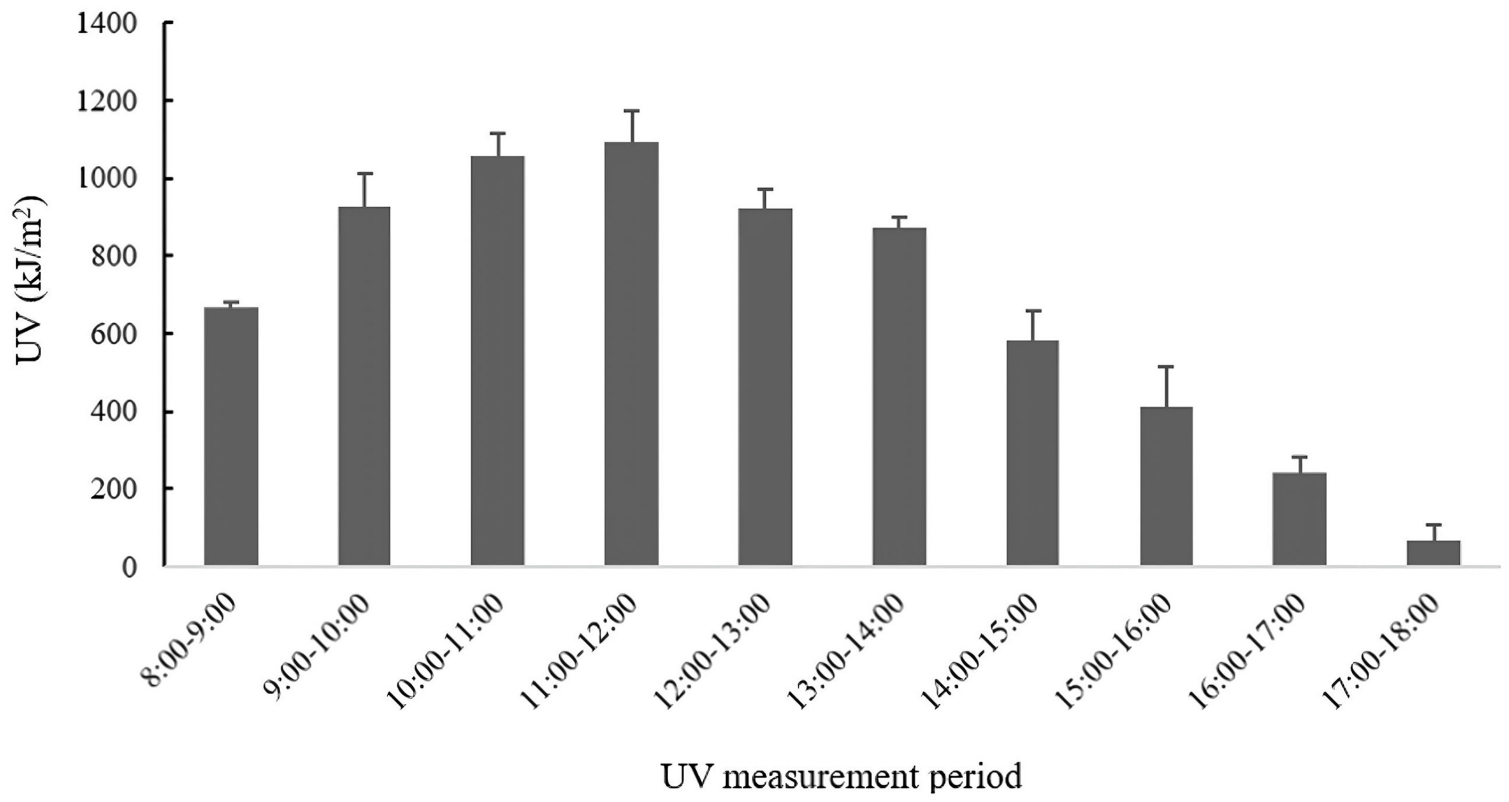
The distribution of UV intensity in different time periods.

Figure 2 shows the average UV intensity in Harbin during different time periods of the day from June to August. The highest UV intensity was recorded from 10:00 to 12:00 (1076.08 ± 64.78 kJ/m^2^), followed by medium intensity from 9:00 to 10:00 and from 12:00 to 14:00 (925.3 ± 62.44 kJ/m^2^), and the lowest intensity from 8:00 to 9:00 and after 14:00 (394.47 ± 233.38 kJ/m^2^). The average UV intensity of different time periods was used to calculate the children's outdoor vitamin D production by UV exposure.

#### 3.1.3 Changes in serum indices and outdoor exercise in outdoor activity intervention

After the intervention, among 61 participants, 53 (86.9%) showed good outdoor activity adherence (serum 25(OH)D_3_: 18.42 ± 3.70 ng/ml) and 8 (13.1%) showed poor adherence (serum 25(OH)D_3_: 18.38 ± 2.14 ng/ml), with no significant difference between the two groups (*P* = 0.975). Compared with baseline, more participants were active during high UV periods (*P* = 0.002), and the mean outdoor activity duration increased by 11 min (*P* = 0.033). However, there were no significant changes in vitamin D production from UV exposure or serum 25(OH)D_3_ concentration after the 8-week intervention (Table 2).

**Table 2:** Serum indices, dietary vitamin D intake, and outdoor exercise before and after outdoor activity intervention^1^.

Variable	Week 0	Week 8	*P*
25(OH)D_3_, ng/ml	18.52 ± 4.07	18.25 ± 3.11	0.499
Energy, kcal/d	1,284.58 ± 419.74	1,346.67 ± 491.37	0.192
Vitamin D from UV, IU/d	328.12 ± 193.62	388.85 ± 250.49	0.682
Vitamin D from diet, IU/d	223.48 ± 46.99	211.51 ± 69.46	0.157
Sun protection, *n* (%)			
Low sun protection	43 (70.5)	40 (65.6)	0.250
High sun protection	18 (29.5)	21 (34.4)	
Outdoor exercise period in summer, *n* (%)			
High UV period	31 (50.8)	42 (68.9) ^*^	0.002
Medium UV period	9 (14.8)	14 (23.0)	
Low UV period	21 (34.4)	5 (8.1)^*^	
Outdoor activity duration, min/d	70.33 ± 31.88	81.48 ± 33.61^*^	0.033

^1^Values are mean ± SD or *n* (%) per group for all other variables. A two-sided *P* < 0.05 was considered statistically significant. ^*^P < 0.05 compared with Week 0.

#### 3.1.4 Association of vitamin D from UV, outdoor activity duration, UV intensity with the change of serum 25(OH)D_3_ concentration of subjects

Linear regression analysis was used to analyze the association of vitamin D from UV, outdoor activity duration, UV intensity with the change of serum 25(OH)D_3_ concentration. After adjustment for age, sex, zBMI, and dietary vitamin D dose, the association of vitamin D from UV, outdoor activity duration, UV intensity with the change in serum 25(OH)D_3_ concentrations was not statistically significant (Table 3). A stratified analysis by sun exposure protection status was further conducted to explore the association between outdoor activity intervention and serum 25(OH)D_3_ levels. No significant intervention effect was observed in either the low sun protection or high sun protection groups. The detailed results are presented in Supplementary Table S1.

**Table 3:** Association of vitamin D from UV, outdoor activity duration, UV intensity with the change of serum 25(OH)D_3_ concentration^1^.

Variable	Vitamin D from UV	*P*	Outdoor activity duration	*P*	UV intensity	*P*
Model 1						
Unstandardized β	1.2E^−5^	0.946	0.001	0.686	−0.638	0.347
Standardized β	0.009		0.056		−0.126	
Model 2						
Unstandardized β	1.49E^−5^	0.934	0.001	0.683	−0.635	0.342
Standardized β	0.011		0.058		−0.125	

^1^Model 1: adjusted for age, sex, zBMI. Model 2: adjusted for age, sex, zBMI, dietary vitamin D dose.

### 3.2 Results from vitamin D supplementation study

#### 3.2.1 Baseline characteristics of subjects by intervention groups

A total of 289 subjects were finally included in this study. The age of children in 800 IU/d vitamin D_3_ group was slightly older than that in the other two groups (*P* = 0.002), and no significant differences were found in other indices among the three groups at baseline (Table 4).

**Table 4:** Baseline characteristics of subjects^1^.

Variable	Placebo (*n* = 106)	400 IU/d vitamin D_3_ (*n* = 93)	800 IU/d vitamin D_3_ (*n* = 90)	*P*
Male, *n* (%)	50 (47.3)	53 (57.0)	53 (58.9)	0.366
Age, years	5.22 ± 1.49	5.15 ± 0.89	5.23 ± 0.77^*^	0.002
zBMI	0.41 ± 1.23	0.34 ± 1.49	0.63 ± 1.24	0.275
Outdoor activity period, *n* (%)				
High UV period	39 (36.7)	12 (13.0)	42 (47.2)	0.070
Medium UV period	56 (53.1)	60 (65.2)	35 (38.9)	
Low UV period	11 (10.2)	21 (21.7)	13 (13.9)	
Outdoor activity duration, min/d	57.40 ± 32.65	42.00 ± 32.89	54.31 ± 31.02	0.231
Vitamin D from diet, IU/d	190.24 ± 53.81	206.88 ± 87.58	220.11 ± 91.05	0.115

^1^Values are mean ± SD or *n* (%) for all variables. A two-sided *P* < 0.05 was considered statistically significant. ^*^P < 0.05 compared with placebo group and 400 IU/d group.

#### 3.2.2 The changes in serum 25(OH)D_3_, ALP and PTH concentrations after vitamin D supplementation

Before the intervention, there were no significant differences in serum 25(OH)D_3_, ALP or PTH concentrations among the three groups (Table 5). After 8 weeks of intervention, serum 25(OH)D_3_ concentration decreased slightly in the placebo group, but increased in the 400 IU/d and 800 IU/d groups. Compared with the placebo group, the two vitamin D-supplemented groups had significantly higher serum 25(OH)D_3_ and ALP concentrations (*P* < 0.001). Their PTH concentrations were significantly lower than that in the placebo group (*P* < 0.001). Dietary vitamin D intake did not change significantly from baseline to post-intervention in any of the three groups (Supplementary Table S2).

**Table 5:** Serum 25(OH)D_3_, ALP and PTH concentration before and after vitamin D supplementation intervention^1^.

Variable	Placebo (*n* = 106)	400 IU/d vitamin D_3_ (*n* = 93)	800 IU/d vitamin D_3_ (*n* = 90)	*P*
25(OH)D_3_, ng/ml				
Baseline	20.26 ± 6.58	18.72 ± 5.98	18.57 ± 4.92	0.076
After intervention	17.09 ± 4.12	28.49 ± 8.60^*^	33.64 ± 9.01^*#^	< 0.001
Total change	−3.26 ± 8.56	9.78 ± 9.06^*^	15.06 ± 9.40^*#^	< 0.001
ALP, U/ml				
Baseline	0.18 ± 0.57	0.20 ± 0.11	0.17 ± 0.05	0.241
After intervention	0.18 ± 0.46	0.21 ± 0.08^*^	0.23 ± 0.08^*#^	< 0.001
Total change	0.001 ± 0.006	0.01 ± 0.10^*^	0.06 ± 0.08^*#^	< 0.001
PTH, pg/ml				
Baseline	34.45 ± 16.60	33.39 ± 14.08	37.55 ± 15.45	0.175
After intervention	39.13 ± 16.13	34.98 ± 11.40^*^	29.84 ± 11.75^*#^	< 0.001
Total change	5.01 ± 17.91	0.74 ± 15.81^*^	−7.92 ± 19.67^*#^	< 0.001

^1^Values are mean ± SD for all variables. A two-sided *P* < 0.05 was considered statistically significant. ^*^Compared with placebo group, *P* < 0.05; ^#^compared with 400 IU/d vitamin D_3_ group, *P* < 0.05. *P* values were adjusted by Tukey.

#### 3.2.3 Association of total vitamin D intake with serum 25(OH)D_3_

**Figure 3:**
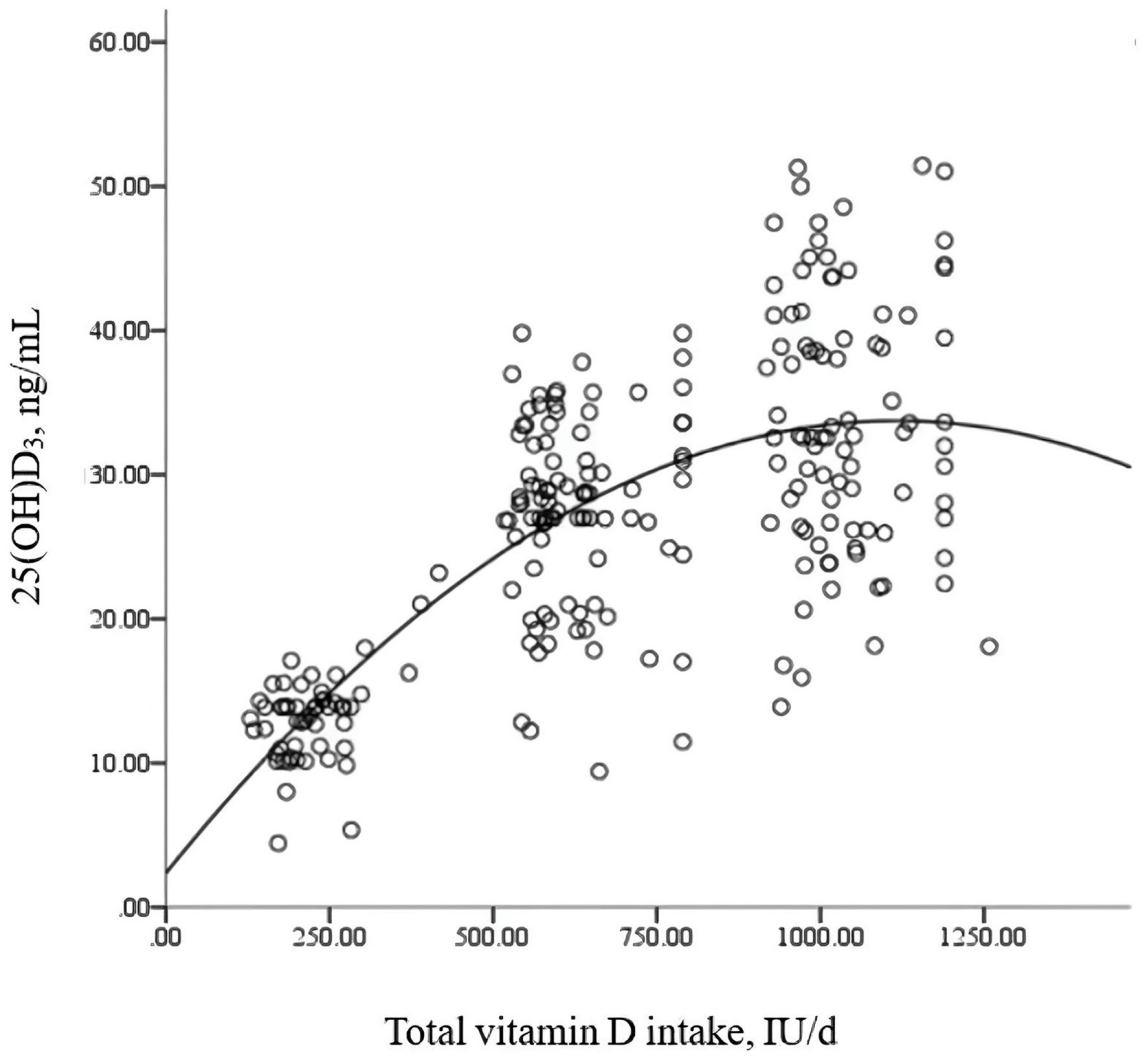
The association of oral vitamin D intake dose with serum 25(OH)D3 concentration in children.

A model for the association of total vitamin D intake (including dietary and supplementation) with serum 25(OH)D_3_ concentration was established. The equation expression was y = 2.38+0.06x-2.52e^−5^x^2^ (x: oral vitamin D intake; y: serum 25(OH)D_3_ concentration; R^2^ = 0.540). In order to maintain the serum 25(OH)D_3_ concentration ≥20 ng/ml and ≥30 ng/ml, the dose of total vitamin D intake should be at least 378.65 IU/d and 733.21 IU/d, respectively (Figure 3). Sensitivity analyses including bootstrap internal validation (1,000 resamples) and multivariable regression adjusting for age, sex, and BMI confirmed the stability of the quadratic model (Supplementary Table S3).

### 3.3 Differences in the change of serum 25(OH)D_3_ concentration among subjects with different SNPs

Two SNPs in the *CYP2R1* (rs1562902, rs10741657) were analyzed. The SNP genotypes distribution is presented in Table 6. In rs1562902, the CC, CT and TT genotypes accounted for 15.1%, 51.4% and 33.5%, respectively. In rs10741657, the AA, AG and GG genotypes accounted for 13.8%, 47.2% and 39.0%, respectively. The distribution of genotypes showed that most children carried mutant genotypes, accounting for more than 80% of the total.

**Table 6:** SNPs genotypes distribution of subjects.

Gene (rs number)	Ref/alt alleles	Alternative allele count	*n*
*CYP2R1* rs1562902	C/T	0	33 (15.1)
		1	112 (51.4)
		2	73 (33.5)
*CYP2R1* rs10741657	A/G	0	30 (13.8)
		1	103 (47.2)
		2	85 (39.0)

Changes of serum 25(OH)D_3_ concentrations in children in the supplementation groups were used to analyze the effect of different SNPs genotypes on the supplementation. Table 7 showed that with the increasing number of variant alleles in rs1562902 and rs10741657, the changes of serum vitamin D concentration were less obvious after supplementation, after adjustment for age, gender, zBMI, serum 25(OH)D_3_ concentration at baseline and vitamin D supplementation dose. Interactions between each SNP genotype and dietary vitamin D intake, vitamin D from UV, as well as outdoor activity duration were further evaluated. No significant gene–environment interactions were observed for changes in serum 25(OH)D_3_ concentration (Supplementary Table S4).

**Table 7:** Changes in serum 25(OH)D_3_ concentrations after vitamin D supplementation among children with different CYP2R1 genotypes^1^.

Gene (rs number)	Ref/alt alleles	Alternative allele count	*n*	Adjusted Δ25(OH)D_3_, ng/ml^1^	*P*
*CYP2R1* rs1562902	C/T	0	26	13.97 ± 1.87	0.288
		1	82	12.42 ± 1.08	
		2	55	10.51 ± 1.33	
*CYP2R1* rs10741657	A/G	0	23	14.12 ± 1.99	0.494
		1	74	12.50 ± 1.16	
		2	66	10.79 ± 1.21	

^1^Adjusted mean ± standard error (SE). Analysis of covariance (ANCOVA) was used to compare mean changes in serum 25(OH)D_3_ across genotype groups, with adjustment for age, sex, zBMI, baseline serum 25(OH)D_3_ concentration, and vitamin D supplementation dose. *P* values were corrected by the Bonferroni multiple comparison test. A two-sided *P* < 0.05 was considered statistically significant.

Children with the CC genotype in rs1562902 and the AA genotype in rs10741657 showed a trend of better supplementation effects, with serum 25(OH)D_3_ concentrations ≥30 ng/mL after receiving an intervention of 400 IU/d vitamin D_3_. In contrast, children with the CT and TT genotypes in rs1562902 and the AG and GG genotypes in rs10741657 needed to receive an intervention of 800 IU/d vitamin D_3_ to reach a serum vitamin D level of over 30 ng/ml (Table 8).

**Table 8:** Serum 25(OH)D_3_ concentrations of children with different SNPs before and after intervention^1^.

Variable	25(OH)D_3_, ng/ml				25(OH)D_3_, ng/ml			
	rs1562902	rs1562902	rs1562902	*P*	rs10741657	rs10741657	rs10741657	*P*
400IU								
	CC (*n* = 16)	CT (*n* = 39)	TT (*n* = 28)		AA (*n* = 14)	AG (*n* = 34)	GG (*n* = 35)	
Baseline	20.38 ± 5.36	17.74 ± 6.46	19.55 ± 5.83	0.613	20.24 ± 5.21	17.98 ± 6.14	19.16 ± 6.37	0.474
After intervention	30.29 ± 10.63	25.74 ± 8.27	25.78 ± 8.44	0.173	30.98 ± 11.48	25.55 ± 8.30	25.93 ± 7.99	0.130
Total change	9.91 ± 13.53	8.00 ± 7.95	6.23 ± 9.57	0.364	10.74 ± 14.40	7.57 ± 7.92	6.77 ± 9.13	0.435
	CC (*n* = 12)	CT (*n* = 44)	TT (*n* = 28)		AA (*n* = 11)	AG (*n* = 40)	GG (*n* = 33)	
800IU								
Baseline	19.60 ± 2.80	18.03 ± 5.25	18.69 ± 5.46	0.264	18.79 ± 3.37	18.68 ± 5.50	18.11 ± 5.00	0.870
After intervention	37.74 ± 11.09	31.61 ± 10.14	31.91 ± 9.94	0.188	36.18 ± 11.13	33.30 ± 9.93	30.52 ± 10.35	0.242
Total change	18.15 ± 11.02	13.58 ± 10.38	13.22 ± 10.77	0.479	17.40 ± 9.99	14.62 ± 10.47	12.41 ± 10.95	0.373

^1^Values are mean ± SD. A two-sided *P* < 0.05 was considered statistically significant. *P* values were adjusted by Tukey.

## 4 Discussion

In this study, we explored the feasibility of increasing UV exposure by increasing outdoor activity and vitamin D supplementation to improve vitamin D deficiency in children in Northeast China. Although UV irradiation of the skin is considered the main source of vitamin D, in the present study it was difficult to obtain adequate vitamin D by increasing outdoor activities. In contrast, supplementation is an effective way to improve vitamin D nutritional status. In order to maintain the serum 25(OH)D_3_ concentration ≥ 30 ng/ml, the dose of total vitamin D intake should be at least 733.21 IU/d. In addition, SNPs may affect the effect of supplementation, and children with CT and TT genotypes in rs1562902 and AG and GG genotypes in rs10741657 showed a trend of lower sensitivity to supplementation.

Although UV irradiation of the skin is considered a good source of vitamin D, vitamin D deficiency is still serious worldwide, especially in children. Multiple factors such as geographical locations, skin exposure area, exposure time and ethnicity can affect the amount of vitamin D produced by UV irradiated skin. Maria's findings showed that in Spain, primary school students aged 6–11 years without sun protection were able to receive a vitamin D dose of 1 100–1 900 IU/d by UV in spring, which was based on theoretical calculations (30), but the dose-response relationship between vitamin D produced by skin and serum 25(OH)D_3_ was not analyzed. A Japanese study with a similar latitude to Northeast China showed that UV exposure was significantly associated with serum 25(OH)D_3_. In summer, increasing UV irradiation time by 10 min could increase 25(OH)D_3_ by 0.47 ng/ml (34). This suggests that although UV irradiation is a good source in theory, it is difficult to obtain sufficient vitamin D by UV alone in practice. In particular, the intensity of UV in winter is significantly weaker than that in summer, and less skin is exposed to UV. In addition, another study demonstrated that to maintain summer 25(OH)D levels during winter, one needed a suberythemal UV dose equal to 1 standard erythema dose (SED) every 2 week to 88% body area. This dose of exposure was equivalent to approximately 10 min of sun exposure at zenith in summertime in Denmark at 56°N (35). However, this effect was not achieved by UV radiation from the outdoors, but by remittance spectroscopy. Meanwhile, latitude is also a key factor. Sunlight-induced vitamin D synthesis becomes ineffective at approximately 40° North latitude, and this limitation extends as latitude increases (12). So far the evidence suggests that production of vitamin D by skin from UV exposure is probably not so easy as we thought. In the present study, we chose the duration of outdoor intervention from June to August, when UV rays were more abundant in Northeast China, and asked the children to increase their outdoor time as much as possible. After the 8-week intervention, the duration and frequency of outdoor activities during the high UV period significantly increased. The amount of vitamin D produced by UV radiation on the skin increased from 328.12 ± 193.62 IU/d to 388.85 ± 250.49 IU/d, but there was no statistical significance in the change of serum 25(OH)D_3_ concentration. Linear regression analysis showed no significant association of vitamin D from UV, outdoor activity duration, UV intensity with the change of serum 25(OH)D_3_ concentration. Even when stratified by sun protection methods, there was no statistically significant association between UV radiation (vitamin D from UV, outdoor activity duration, and UV intensity) and the changes in serum 25(OH)D_3_. At baseline, children already spent an average of 70 min outdoors daily. Considering the daily schedule of Chinese kindergartens (including class and nap times), further extending outdoor time is practically difficult. Moreover, after returning home from kindergarten, children are exposed to off-peak UV intensity, which further limits effective vitamin D synthesis. In addition, Northeast China is located at a relatively high latitude, where naturally low UV intensity directly limits skin vitamin D production, especially during winter. Therefore, relying solely on increased outdoor UV exposure to improve vitamin D status may not be a practically feasible strategy for children living in high-latitude regions.

Due to the difficulty of increasing the time and effectiveness of outdoor activities, the vitamin D levels of subjects were still insufficient even in summer in the present study. In this case without timely and effective intervention, the vitamin D nutritional status of children in winter would only further deteriorate. Therefore, it is necessary to ensure enough intake of vitamin D through oral administration. In order to maintain adequate vitamin D nutritional status in children, governments or relevant agencies have formulated recommended intake levels. According to the 2024 American Endocrine Society guidelines, the recommended dietary reference intake for children aged 118 years should be 600 IU/d (36). For children aged 4–10 in Poland who do not receive regular exposure to sunshine, it is recommended that they consume 600–1000 IU/d (37). In China, the dietary reference intakes (DRIs) for children should be 400 IU/d, while some experts recommend that the intake of vitamin D should be increased to 600 IU/d (38). However, vitamin D deficiency in children has not been alleviated even with this recommended intake. Compared with surveys from 2010–2012, the deficiency rate rose from 17.36% to 21.29% in 2016–2017. Even in southern regions with lower latitudes and sufficient sunshine, the rate remained as high as 12.67% (6). This suggests that, given Chinese children's current lifestyle, dietary vitamin D alone may be insufficient to improve their nutritional status, thus additional vitamin D supplementation is necessary to elevate their serum vitamin D levels. Some studies have also explored the effect of vitamin D supplementation among children. In an American randomized controlled trial, children aged 7 months to 10 years received 400 or 1,000 IU/day vitamin D for 6 months. Serum 25(OH)D levels increased from 65.5 ± 16.5 to 76.8 ± 18.0 nmol/L in the 400 IU group and from 58.3 ± 17.0 nmol/L to 85.5 ± 22.8 nmol/L in the 1,000 IU group (39). A supplementation trial conducted in Mexico showed that 1000 IU/d vitamin D for 3 months increased serum 25(OH)D_3_ concentration from 59.8 ± 1.88 nmol/L to 79.5 ± 2.18 nmol/L in children aged 12 to 30 months (20). In an 18-month intervention study of children aged 9-15 years in Zhejiang Province, China, serum 25(OH)D_3_ concentration increased from 12.77 ± 3.01 ng/ml to 17.34 ± 3.78 ng/mL after 400 IU/d supplementation (40). In this study, children were divided into three groups and were administered vitamin D_3_ at placebo, 400 IU/d, and 800 IU/d respectively, for an 8-week intervention period, to explore the optimal oral vitamin D dosage for improving the vitamin D nutritional status. Our results showed that after 8 weeks of supplementation, the serum 25(OH)D_3_ concentration increased from 18.38 ± 5.98 ng/ml to 27.54 ± 6.52 ng/ml in children supplemented with 400 IU/d, and increased from 18.57 ± 4.92 ng/ml to 33.64 ± 9.01 ng/ml in children supplemented with 800 IU/d, indicating a greater improvement in vitamin D nutrition levels in the high-dose group. In addition, there were also dose-responses in the changes of serum PTH and ALP concentrations among the groups. Although oral supplementation can significantly improve vitamin D levels, the efficacy varies across countries and even among different regions within the same country. The results of this study suggest that there may be certain differences in the intervention responses to vitamin D supplementation in children of different ages and from different regions, and further study with more comprehensive design should be carried out.

To determine the optimal oral dose, it is ideal to consider dietary intake first. Many scholars have conducted research on the relationship between dietary vitamin D and serum vitamin D level. A study has found that white adolescents in the northeastern United States required 400 and 1,200 IU/d vitamin D from diet and supplements during winter to maintain serum 25(OH)D concentrations > 10–20 ng/ml, respectively (41). Swedish children required 240 and 800 IU/d vitamin D from diet and supplements to maintain serum 25(OH)D concentrations > 10–20 ng/ml, respectively (42). Females aged 11–14 years in the northeastern Denmark and Finland required 332 and 744 IU/d vitamin D from diet and supplements during the winter to maintain serum 25(OH)D concentrations > 10–20 ng/mL, respectively (43). There are differences in the existing research results. Taking into account differences in ethnicity, living location and baseline serum 25(OH)D_3_ concentration, the required oral vitamin D intake varies according to serum vitamin D status. However, there is currently no similar report in China. Therefore, we analyzed the dose-response relationship between total vitamin D intake and serum 25(OH)D_3_. Our results showed that children needed 378.65 IU/d and 733.21 IU/d to ensure serum 25(OH)D_3_ ≥20 ng/ml and ≥30 ng/ml, respectively. In this study, the total intake of vitamin D included dietary and supplemental doses. Dietary surveys in our study showed that the average level of dietary vitamin D was only 267.27 ± 96.02 IU/d, which was insufficient to meet nutritional requirements. These findings indicate that dietary vitamin D alone is inadequate to achieve optimal vitamin D status in children, and additional oral supplementation is therefore indispensable. Notably, our estimated intakes are slightly higher than current Chinese expert consensus. Further large-scale, well-designed studies are needed to establish evidence-based recommendations for children across different regions in China.

Some genetic polymorphisms can also affect vitamin D levels. *CYP2R1* is the major hydroxylase in the liver that carries the hydroxylation of vitamin D to 25(OH)D (44). People with vitamin D deficiency had a higher frequency of SNP rs10741657 in *CYP2R1*, namely AG and GG genotypes (45). In a study involving Arabs, people with CC genotype in *CYP2R1* rs7116978 were more likely to have adequate vitamin D levels, and people with CC genotype in rs7116978 showed a higher response to vitamin D supplementation (26). Afsane Bahrami's study showed that subjects with the AA genotype in *CYP2R1* rs10766197 had more significant changes in vitamin D levels after taking vitamin D supplementation (46). These studies suggest that SNPs are not only closely related to the synthesis and metabolism of vitamin D, but also may affect the effectiveness of vitamin D supplementation. In this study, we explored whether SNPs also affect the effect of vitamin D supplementation, with focus on rs1562902 and rs10741657. Most of the subjects in this study had mutant genotypes. In rs1562902, the CT and TT genotypes accounted for 51.4% and 33.5%, respectively. In rs10741657, the AG and GG genotypes accounted for 47.2% and 39.0%, respectively. Results showed that children with CT/TT (rs1562902) and AG/GG (rs10741657) were less sensitive to supplementation. Children with CC (rs1562902) and AA (rs10741657) reached ≥30 ng/mL after 400 IU/d for 8 weeks. In contrast, those with alternative alleles needed 800 IU/d to reach the same level. From a precise nutrition perspective, children with different SNP genotypes and different dietary vitamin D intakes differ in their sensitivity to vitamin D supplementation, warranting further research on genotype-specific vitamin D requirements.

This study has several strengths. First, it is one of the few studies in Northeast China that simultaneously considered outdoor UV exposure, dietary intake, supplementation, and genetic polymorphisms in relation to children's vitamin D status. Second, we provided a region-specific estimate of the total vitamin D intake required to improve children's serum vitamin D nutritional status, which can serve as a preliminary reference for local nutrition guidelines. Third, although these genetic-related differences are only preliminary results, they have laid the foundation for further research on precise nutrition for children living in high-latitude regions.

It is important to note that there are some limitations. This is a single-center, single-blind study, which may limit its external validity. The two intervention groups were implemented in different seasons and populations, and were not designed for direct statistical comparison. We did not measure some potential confounding factors (such as skin type, air pollution, previous sun exposure), and evaluated compliance through parent reports and capsule counts, which may introduce bias. The ultraviolet exposure dose was estimated by a fixed-position measuring instrument and assumed the skin exposure area, which may lead to inaccuracy. The sample size was relatively small, especially in terms of genetic analysis, where we only detected two single nucleotide polymorphisms (SNPs), and did not correct for multiple comparisons. Vitamin D dosages were not determined based on genotype. Finally, the 8-week follow-up period cannot assess long-term efficacy or safety. Therefore, our conclusions should be interpreted with caution and require confirmation through longer follow-up in larger-scale, multi-center, double-blind trials.

## 5 Conclusion

In conclusion, under the conditions of this study, increasing outdoor activities did not significantly improve vitamin D status, limited by lifestyles and geographic location. Oral supplementation is a viable method to improve vitamin D deficiency, and a total intake of at least 733.21 IU/d (including dietary intake and supplemental intake) can help maintain the serum 25(OH)D_3_ concentration at ≥30 ng/mL. The SNPs related to vitamin D metabolism may affect the effectiveness of supplementation. This study provides valuable information for improving vitamin D levels in children in Northeast China from the perspective of practical and precision nutrition.

## Data Availability

The datasets presented in this article are available from the corresponding author upon reasonable request. Requests to access the datasets should be directed to Lixin Na, E-mail: nalixin2003@163.com.

## References

[1] Kupisz-Urbanska M Pludowski P Marcinowska-Suchowierska E. Vitamin D Deficiency in older patients-problems of sarcopenia, drug interactions, management in deficiency. Nutrients. (2021) 13:1247. doi: 10.3390/nu13041247 33920130 PMC8069639

[2] Liu W Hu J Fang Y Wang P Lu Y Shen N. Vitamin D status in Mainland of China: A systematic review and meta-analysis. EClinicalMedicine. (2021) 38:101017. doi: 10.1016/j.eclinm.2021.101017 34308318 PMC8283334

[3] Dunlop E Pham NM Van Hoang D Mazahery H Neo B Shrapnel J . A systematic review and meta-analysis of circulating 25-hydroxyvitamin D concentration and vitamin D status worldwide. J Public Health. (2025) 47:e520–9. doi: 10.1093/pubmed/fdaf080 40652566 PMC12670000

[4] Siddiqee MH Bhattacharjee B Siddiqi UR Rahman MM. High burden of hypovitaminosis D among the children and adolescents in South Asia: a systematic review and meta-analysis. J Health Popul Nutr. (2022) 41:10. doi: 10.1186/s41043-022-00287-w 35300737 PMC8929474

[5] Arshad S Zaidi SJA. Vitamin D levels among children, adolescents, adults, and elders in Pakistani population: a cross-sectional study. BMC Public Health. (2022) 22:2040. doi: 10.1186/s12889-022-14526-6 36348325 PMC9641307

[6] Hu Y Jiang S Lu J Yang Z Yang X Yang L. Vitamin D Status for Chinese Children and Adolescents in CNNHS 2016-2017. Nutrients. (2022) 14:4928. doi: 10.3390/nu14224928 36432613 PMC9693967

[7] Herdea A Marie H Ionescu A Sandu DM Pribeagu ST Ulici A. Vitamin D Deficiency-A public health issue in Children. Children. (2024) 11:1061. doi: 10.3390/children11091061 39334594 PMC11429966

[8] Van de Walle L Vandenplas Y Toelen J Raaijmakers A. Vitamin D Status in Belgian children: a regional study. Nutrients. (2024) 16:657. doi: 10.3390/nu16050657 38474785 PMC10935432

[9] Setayesh L Casazza K Moradi N Mehranfar S Yarizadeh H Amini A . Association of vitamin D-binding protein and vitamin D(3) with insulin and homeostatic model assessment (HOMA-IR) in overweight and obese females. BMC Res Notes. (2021) 14:193. doi: 10.1186/s13104-021-05608-6 34011380 PMC8136187

[10] Weller RB. Sunlight: Time for a rethink? J Invest Dermatol. (2024) 144:1724–32. doi: 10.1016/j.jid.2023.12.027 38661623

[11] Latoch E Kozlowski K Kononczuk K Zelazowska-Rutkowska B Tomczuk-Ostapczuk M Krawczuk-Rybak M . Vitamin D deficiency and carotid media-intima thickness in childhood cancer survivors. Nutrients. (2023) 15:2333. doi: 10.3390/nu15102333 37242216 PMC10220745

[12] Kift RC Webb AR. Globally estimated UVB exposure times required to maintain sufficiency in Vitamin D levels. Nutrients. (2024) 16:1489. doi: 10.3390/nu16101489 38794727 PMC11124381

[13] Elliott TM Gordon LG Webb A Kift R Foeglein A Neale RE. Making the sunshine vitamin - How much sun exposure is needed to maintain 25-hydroxy vitamin D concentration? Photochem Photobiol. (2024) 100:746–55. doi: 10.1111/php.13854 37691266

[14] Thomas DT Erdman KA Burke LM. American college of sports medicine joint position statement. Nutrition and athletic performance. Med Sci Sports Exerc. (2016) 48:543–68. doi: 10.1249/MSS.00000000000000852 26891166

[15] Larson-Meyer DE Willis KS. Vitamin D and athletes. Curr Sports Med Rep. (2010) 9:220–6. doi: 10.1249/JSR.0b013e3181e7dd45 20622540

[16] Beadel H Sandiford NA. Higher prevalence of vitamin D deficiency among arthroplasty patients presenting in winter. J Orthop Surg Res. (2025) 20:1050. doi: 10.1186/s13018-025-06482-9 41316301 PMC12664226

[17] Abdi SAH Ali A Sayed SF Nagarajan S Abutahir Alam P . Sunscreen ingredient octocrylene's potency to disrupt vitamin D Synthesis. Int J Mol Sci. (2022) 23:154. doi: 10.3390/ijms231710154 36077552 PMC9456232

[18] Wang LL Wang HY Wen HK Tao HQ Zhao XW. Vitamin D status among infants, children, and adolescents in southeastern China. J Zhejiang Univ Sci B. (2016) 17:545–52. doi: 10.1631/jzus.B1500285 27381731 PMC4940630

[19] Moon RJ Curtis EM Cooper C Davies JH Harvey NC. Vitamin D supplementation: are multivitamins sufficient? Arch Dis Child. (2020) 105:791–3. doi: 10.1136/archdischild-2018-316339 30804007 PMC7115846

[20] Flores-Aldana M Rivera-Pasquel M Garcia-Guerra A Perez-Cortes JG Barcena-Echegollen JE. Effect of Vitamin D Supplementation on (25(OH)D) status in children 12-30 months of age: a randomized clinical Trial. Nutrients. (2023) 15:2756. doi: 10.3390/nu15122756 37375660 PMC10304884

[21] Ganmaa D Bromage S Khudyakov P Erdenenbaatar S Delgererekh B Martineau AR. Influence of Vitamin D supplementation on growth, body composition, and pubertal development among school-aged children in an area with a high prevalence of vitamin d deficiency: a randomized clinical trial. JAMA Pediatr. (2023) 177:32–41. doi: 10.1001/jamapediatrics.2022.4581 36441522 PMC9706398

[22] Asghari G Yuzbashian E Najd-Hassan-Bonab L Mirmiran P Daneshpour MS Azizi F. Association of rs2282679 polymorphism in vitamin D binding protein gene (GC) with the risk of vitamin D deficiency in an iranian population: season-specific vitamin D status. BMC Endocr Disord. (2023) 23:217. doi: 10.1186/s12902-023-01463-7 37814286 PMC10563356

[23] Mydlarova Blascakova M Lorinczova Z Anderkova L Czerwinska-Ledwig O Mikulova L Hrusovska H . Relationship between vitamin D receptor gene bsmi polymorphism and 25-hydroxyvitamin D total levels in slovak postmenopausal women with reduced bone mineral density. Genes. (2025) 16:337. doi: 10.3390/genes16030337 40149488 PMC11941902

[24] Zhou W Wang P Bai Y Zhang Y Shu J Liu Y. Vitamin D metabolic pathway genes polymorphisms and vitamin D levels in association with neonatal hyperbilirubinemia in China: a single-center retrospective cohort study. BMC Pediatr. (2023) 23:275. doi: 10.1186/s12887-023-04086-y 37259065 PMC10230706

[25] Kelishadi R Heidari-Beni M Akbarian SA Hasan Tajadini M Haghjooy Javanmard S. Genetic variation in cytochrome P450 2R1 and vitamin d binding protein genes are associated with vitamin d deficiency in adolescents. Int J Vitam Nutr Res. (2020) 90:339–45. doi: 10.1024/0300-9831/a000632 32517587

[26] Tomei S Singh P Mathew R Mattei V Garand M Alwakeel M . The Role of Polymorphisms in Vitamin D-Related Genes in Response to Vitamin D Supplementation. Nutrients. (2020) 12:2608. doi: 10.3390/nu12092608 32867112 PMC7551134

[27] Liu J Fu L Jin S Jia Y Zhang J Sun C . Vitamin D status in children and its association with glucose metabolism in northern China: a combination of a cross-sectional and retrospective study. BMJ Open. (2022) 12:e061146. doi: 10.1136/bmjopen-2022-061146 36446458 PMC9710338

[28] Group WHOMGRS. WHO Child Growth Standards based on length/height, weight and age. Acta Paediatr Suppl. (2006) 450:76–85. doi: 10.1111/j.1651-2227.2006.tb02378.x 16817681

[29] de Onis M Onyango AW Borghi E Siyam A Nishida C Siekmann J. Development of a WHO growth reference for school-aged children and adolescents. Bull World Health Organ. (2007) 85:660–7. doi: 10.2471/blt.07.043497 18026621 PMC2636412

[30] Serrano MA. Contribution of sun exposure to the vitamin D dose received by various groups of the Spanish population. Sci Total Environ. (2018) 619-620:545-551. doi: 10.1016/j.scitotenv.2017.11.036 29156273

[31] Godar DE Pope SJ Grant WB Holick MF. Solar UV doses of adult Americans and vitamin D(3) production. Dermatoendocrinol. (2011) 3:243–50. doi: 10.4161/derm.3.4.15292 22259652 PMC3256341

[32] Agriculture USDo. Agricultural Research Service USDA National Nutrient Database for Standard Reference, Release 28. (2015). Available online at: http://www.ars.usda.gov/ba/bhnrc/ndl (Accessed July 15, 2026).

[33] Denova-Gutierrez E Munoz-Aguirre P Lopez D Flores M Medeiros M Tamborrel N . Low serum vitamin d concentrations are associated with insulin resistance in mexican children and adolescents. Nutrients. (2019) 11:2109. doi: 10.3390/nu11092109 31491877 PMC6770751

[34] Asakura K Etoh N Imamura H Michikawa T Nakamura T Takeda Y . Vitamin D Status in Japanese Adults: relationship of serum 25-hydroxyvitamin d with simultaneously measured dietary vitamin d intake and ultraviolet ray exposure. Nutrients. (2020) 12:743. doi: 10.3390/nu12030743 32168939 PMC7146414

[35] Bogh MK Schmedes AV Philipsen PA Thieden E Wulf HC A. small suberythemal ultraviolet B dose every second week is sufficient to maintain summer vitamin D levels: a randomized controlled trial. Br J Dermatol. (2012) 166:430–3. doi: 10.1111/j.1365-2133.2011.10697.x 22013924

[36] Demay MB Pittas AG Bikle DD Diab DL Kiely ME Lazaretti-Castro M . Vitamin D for the prevention of disease: an endocrine society clinical practice guideline. J Clin Endocrinol Metab. (2024) 109:1907–47. doi: 10.1210/clinem/dgae290 38828931

[37] Pludowski P Kos-Kudla B Walczak M Fal A Zozulinska-Ziolkiewicz D Sieroszewski P . Guidelines for preventing and treating vitamin d deficiency: a 2023 update in Poland. Nutrients. (2023) 15:695. doi: 10.3390/nu15030695 36771403 PMC9920487

[38] Tang S. Expert consensus on the evaluation and improvement of vitamin D nutritional status. J Chin Soc Health Manag. (2023) 17: 245–252. doi: 10.3760/cma.j.cn115624-20230105-00009

[39] Simpson CA Zhang JH Vanderschueren D Fu L Pennestri TC Bouillon R . 25-OHD response to vitamin D supplementation in children: effect of dose but not GC haplotype. Eur J Endocrinol. (2021) 185:333–42. doi: 10.1530/EJE-21-0349 34128826 PMC8284876

[40] Zhang R Muyiduli X Su D Zhou B Fang Y Jiang S . Effect of Low-Dose Vitamin D Supplementation on Serum 25(OH)D in School Children and White-Collar Workers. Nutrients. (2017) 9:505. doi: 10.3390/nu9050505 28513555 PMC5452235

[41] Smith TJ Tripkovic L Damsgaard CT Molgaard C Ritz C Wilson-Barnes SL . Estimation of the dietary requirement for vitamin D in adolescents aged 14-18 y: a dose-response, double-blind, randomized placebo-controlled trial. Am J Clin Nutr. (2016) 104:1301–9. doi: 10.3945/ajcn.116.138065 27655438

[42] Ohlund I Lind T Hernell O Silfverdal SA Karlsland Akeson P. Increased vitamin D intake differentiated according to skin color is needed to meet requirements in young Swedish children during winter: a double-blind randomized clinical trial. Am J Clin Nutr. (2017) 106:105–12. doi: 10.3945/ajcn.116.147108 28615261

[43] Cashman KD FitzGerald AP Viljakainen HT Jakobsen J Michaelsen KF Lamberg-Allardt C . Estimation of the dietary requirement for vitamin D in healthy adolescent white girls. Am J Clin Nutr. (2011) 93:549–55. doi: 10.3945/ajcn.110.006577 21270380

[44] Iriani A Rachman A Fatina MK Gemilang RK Trisnandi A Muskananfola FV . Vitamin D status, vitamin D receptor, CYP2R1, and CYP24A1 profiles in children. Front Nutr. (2024) 11:1394367. doi: 10.3389/fnut.2024.1394367 38912300 PMC11190155

[45] Rahman A Abu-Farha M Channanath A Hammad MM Anoop E Chandy B . Single nucleotide polymorphisms in vitamin D binding protein and 25-hydroxylase genes affect vitamin D levels in adolescents of Arab ethnicity in Kuwait. Front Endocrinol. (2023) 14:1257051. doi: 10.3389/fendo.2023.1257051 37929021 PMC10623322

[46] Bahrami A Mehramiz M Ghayour-Mobarhan M Bahrami-Taghanaki H Sadeghi Ardekani K Tayefi M . A genetic variant in the cytochrome P450 family 2 subfamily R member 1 determines response to vitamin D supplementation. Clin Nutr. (2019) 38:676–81. doi: 10.1016/j.clnu.2018.03.018 29752008

