# Supplementary material for: Intervention methods and influencing factors of vitamin D deficiency in Northeast China: a self-controlled trial and randomized controlled trial study
Source: Front Nutr. 2026 Aug 3;13:1778526. doi: 10.3389/fnut.2026.1778526 (PMC13477899; doi:10.3389/fnut.2026.1778526)
Supplement: Supplementary file 1 [file Table_1.docx]

**Supplemental Table S1.** Stratified analysis of association of vitamin D from UV, outdoor activity duration, UV intensity with the change of serum 25(OH)D_3_ concentration, by sun exposure protection status ^1^.

| **Variable** | **vitamin D from UV** | ***P*** | **Outdoor activity duration** | ***P*** | **UV intensity** | ***P*** |
| --- | --- | --- | --- | --- | --- | --- |
| Low sun protection |  |  |  |  |  |  |
| Model 1 |  |  |  |  |  |  |
| Unstandardized β | 0.003 | 0.095 | 0.021 | 0.203 | -7.275 E^-5^ | 0.501 |
| Standardized β | 0.254 |  | 0.206 |  | -0.110 |  |
| Model 2 |  |  |  |  |  |  |
| Unstandardized β | 0.001 | 0.552 | 0.022 | 0.177 | -6.246 E^-5^ | 0.565 |
| Standardized β | 0.094 |  | 0.223 |  | -0.094 |  |
| High sun protection |  |  |  |  |  |  |
| Model 1 |  |  |  |  |  |  |
| Unstandardized β | -0.002 | 0.569 | 0.017 | 0.618 | 1.18 E^-5^ | 0.469 |
| Standardized β | -0.145 |  | 0.145 |  | 0.158 |  |
| Model 2 |  |  |  |  |  |  |
| Unstandardized β | -0.002 | 0.608 | 0.016 | 0.651 | 1.39E^-4^ | 0.419 |
| Standardized β | -0.134 |  | 0.135 |  | 0.218 |  |

¹Model 1: Adjusted for age, sex, zBMI. Model 2: Adjusted for age, sex, zBMI, dietary vitamin D dose.

**Supplemental Table S2.** Dietary vitamin D intake at baseline and after intervention among the three groups^1^.

| **Variable** | **Baseline, IU/d** | **Post-intervention**, **IU/d** | ***P*** |
| --- | --- | --- | --- |
| Placebo | 190.22±53.81 | 213.44±51.2 IU/d | 0.720 |
| 400 IU/d vitamin D_3_ | 206.88±87.58 | 205.50±57.95 | 0.631 |
| 800 IU/d vitamin D_3_ | 220.11±91.05 | 214.66±64.36 | 0.143 |

^1^Values are mean ± SD for all variables. A two-sided *P* < 0.05 was considered statistically significant.

**Supplemental Table S3. Sensitivity analyses for the quadratic dose‑response model**

| Analysis | Quadratic term coefficient | 95% CI | R² |
| --- | --- | --- | --- |
| Bootstrap validation^1^ | −2.52×10⁻⁵ | −3.62e⁻⁵ - −1.42e⁻⁵ | 0.545 |
| Multivariable regression ^2^ | −2.45×10⁻⁵ | −3.02e⁻⁵ - −1.88e⁻⁵ | 0.570 |

^1^ Bootstrap results were based on 1000 resamples with bias-corrected accelerated (BCa) confidence intervals.

^2^ Multivariable regression retained the quadratic terms (oral vitamin D intake and oral vitamin D intake squared) while adjusting for age, sex, and zBMI. The quadratic term was significant in both analyses (p < 0.001).

**Supplemental Table S4.** Interactions between each SNP genotype and dietary vitamin D intake, vitamin D from UV and outdoor activity duration ^1^

| **Variable** | ***F* value** | **Degrees of Freedom (*df*)** | ***P*** |
| --- | --- | --- | --- |
| rs1562902 |  |  |  |
| rs1562902×vitamin D from diet | 1.335 | 4 | 0.460 |
| rs1562902×vitamin D from UV | 0.517 | 4 | 0.723 |
| rs1562902× outdoor activity duration | 0.082 | 4 | 0.988 |
| rs10741657 |  |  |  |
| rs10741657×vitamin D from diet | 1.246 | 4 | 0.293 |
| rs10741657×vitamin D from UV | 0.421 | 4 | 0.793 |
| rs10741657× outdoor activity duration | 0.087 | 4 | 0.986 |

¹Model 1: Adjusted for age, sex, zBMI. Model 2: Adjusted for age, sex, zBMI, dietary vitamin D dose.
